# Supplementary material for: The DEPTQ+ Experiment: Leveling the DEPT Signal Intensities and Clean Spectral Editing for Determining CHn Multiplicities
Source: Molecules. 2021 Jun 8;26(12):3490. doi: 10.3390/molecules26123490 (PMC8228129; doi:10.3390/molecules26123490)
Supplement: Supplementary file 1 [file molecules-26-03490-s001.zip › molecules-1231661-supplementary.pdf]

molecules

The DEPTQ+ Experiment: Leveling the DEPT Signal Intensities and Clean Spectral Editing for Determining CH_n Multiplicities

Peter Bigler*, Camilo Melendez, Julien Furrer*

Departement für Chemie, Biochemie und Pharmazie, Universität Bern, Freiestrasse 3, CH-3012 Bern, Switzerland

Supporting Information

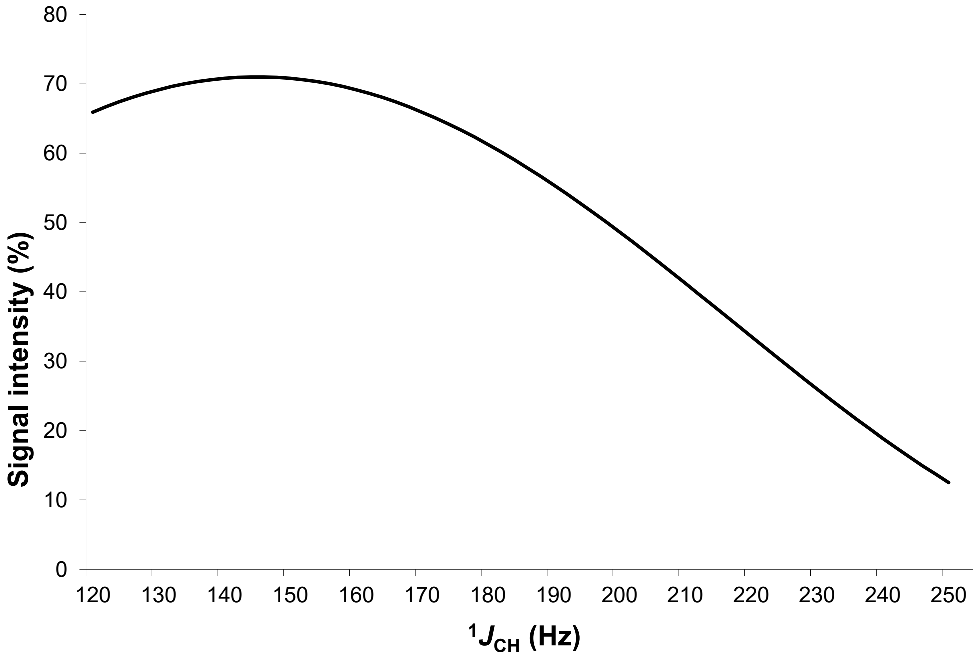

Figure S1. DEPTQ135$_{90}$: theoretical amplitude of a CH group as a function of the $^1J_{CH}$ coupling constant. The equation used for obtaining the theoretical amplitude is: $0.71\sin^2(\pi J_{CH}\delta)$. The delay δ was set to 3.45 ms, adjusted for a coupling constant $^1J_{CH}$ of 145 Hz.

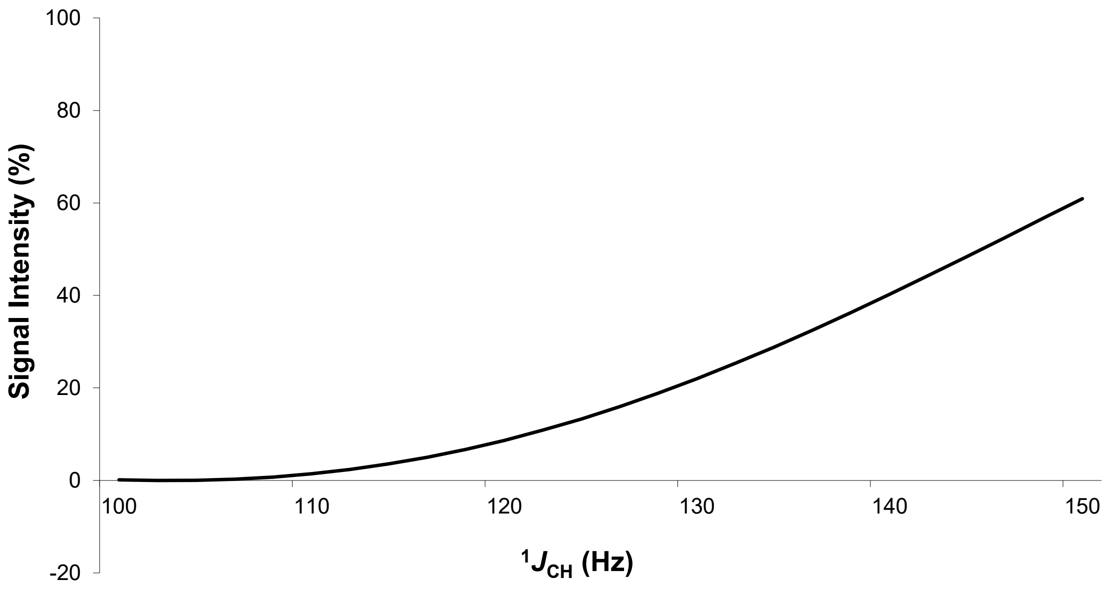

Figure S2. DEPTQ135$_{90}$: theoretical amplitude of CH_3 groups as a function of the $^1J_{CH}$ coupling constant. The equation used for obtaining the theoretical amplitudes is: $0.53\ \sin^2(2\pi J_{CH}\delta)\cos^2(\pi J_{CH}\delta) - 0.75\ \sin^2(2\pi J_{CH}\delta)\sin^2(\pi J_{CH}\delta) + 1.06\ \sin^6(\pi J_{CH}\delta)$. The delay δ was set to 2.70 ms, adjusted for a coupling constant $^1J_{CH}$ of 185 Hz, to simulate that the full range of $^1J_{CH}$ coupling constant [100 – 250 Hz] is considered.

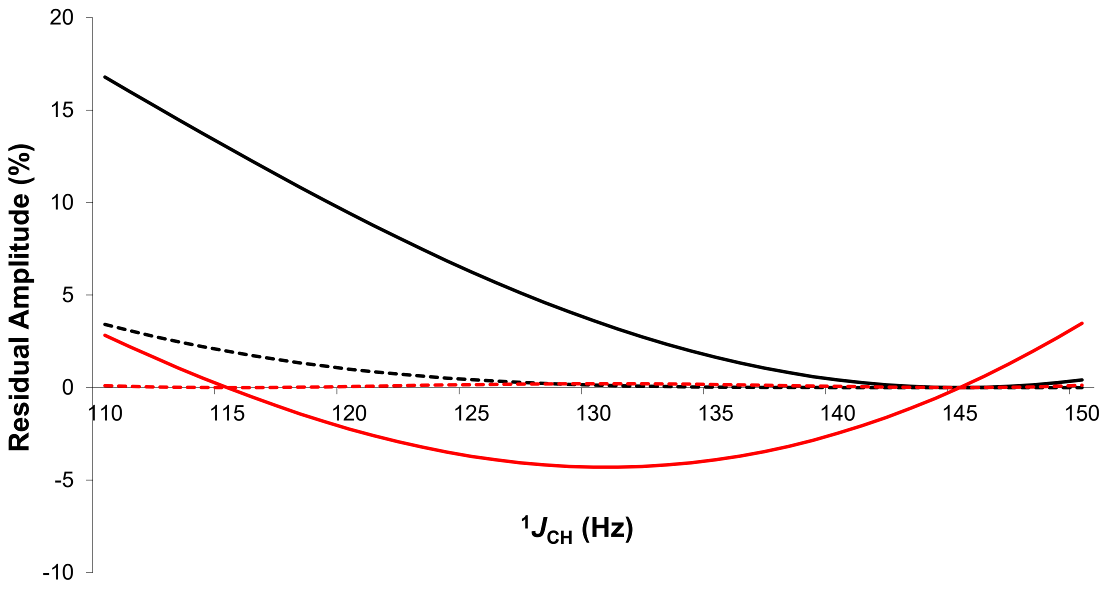

Figure S3. Black lines: DEPTQ90$_{45}$, red lines: DEPTQ$^+$90$_{45}$: theoretical residual amplitude of CH_2 and CH_3 groups as a function of the $^1J_{CH}$ coupling constant. Plain lines: CH_2 groups, dashed lines: CH_3 groups. The equations used for obtaining the theoretical amplitudes are: CH_2 groups: $0.35\ \sin^2(2\pi J_{CH}\delta)$ for DEPTQ and $0.71\ \sin(\pi J_{CH}\delta_1)\cos(\pi J_{CH}\delta_2)\sin(2\pi J_{CH}\delta_3)$ for DEPTQ$^+$, CH_3 groups: $2.11\ \sin^2(\pi J_{CH}\delta)\cos^4(\pi J_{CH}\delta)$ for DEPTQ and $2.11\ \sin(\pi J_{CH}\delta_1)\cos^2(\pi J_{CH}\delta_2)\sin(\pi J_{CH}\delta_3)\cos^2(\pi J_{CH}\delta_3)$ for DEPTQ$^+$. The $^1J_{CH}$ coupling constant range considered is [110 – 185 Hz], to simulate a molecule

possessing usual $^1J_{CH}$ coupling constants. For the DEPTQ90_{45} experiment, the delay δ was set to 3.45 ms, adjusted for a coupling constant $^1J_{CH}$ of 145 Hz. For the DEPTQ$^+90_{45}$ experiment, the delay δ_1 was set to 2.95 ms, adjusted for a coupling constant $^1J_{CH}$ of 170 Hz, δ_2 was set to 4.35 ms, adjusted for a coupling constant $^1J_{CH}$ of 115 Hz, and δ_3 was set to 3.45 ms, adjusted for a coupling constant $^1J_{CH}$ of 145 Hz.

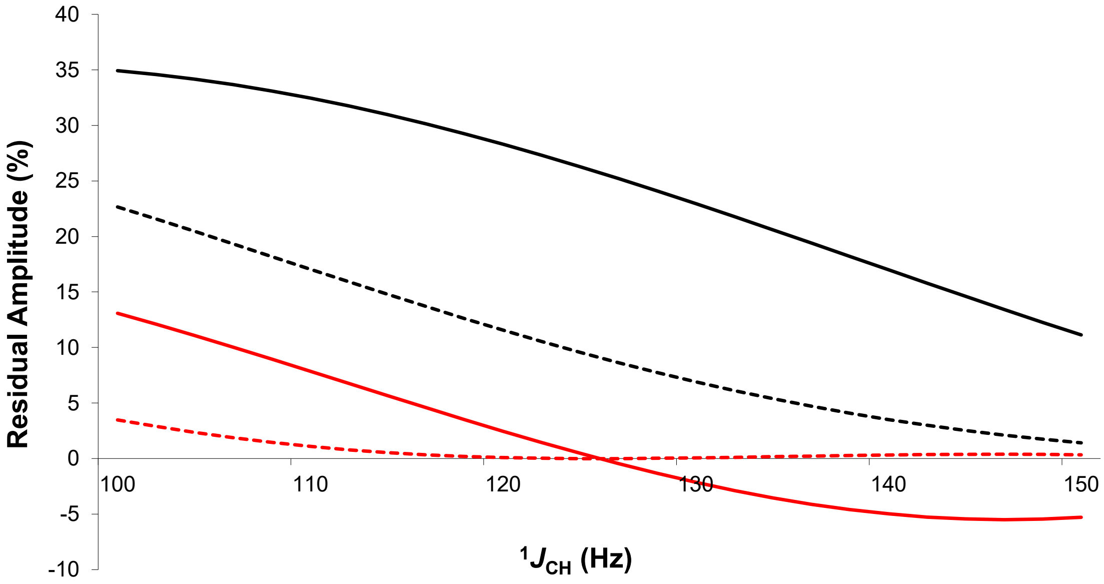

Figure S4. Black lines: DEPTQ90_{45}, red lines: DEPTQ$^+90_{45}$: theoretical residual amplitude of CH_2 and CH_3 groups as a function of the $^1J_{CH}$ coupling constant. Plain lines: CH_2 groups, dashed lines: CH_3 groups. The equations used for obtaining the theoretical amplitudes are: CH_2 groups: 0.35 $\sin^2(2\pi J_{CH}\delta)$ for DEPTQ and 0.71 $\sin(\pi J_{CH}\delta_1)\cos(\pi J_{CH}\delta_2)\sin(2\pi J_{CH}\delta_3)$ for DEPTQ$^+$, CH_3 groups: 2.11 $\sin^2(\pi J_{CH}\delta)\cos^4(\pi J_{CH}\delta)$ for DEPTQ and 2.11 $\sin(\pi J_{CH}\delta_1)\cos^2(\pi J_{CH}\delta_2)\sin(\pi J_{CH}\delta_3)\cos^2(\pi J_{CH}\delta_3)$ for DEPTQ$^+$. The $^1J_{CH}$ coupling constant range considered is [110 – 250 Hz], to simulate a molecule possessing all types of $^1J_{CH}$ coupling constants. For the DEPTQ90_{45} experiment, the delay δ was set to 2.70 ms, adjusted for a coupling constant $^1J_{CH}$ of 185 Hz. For the DEPTQ$^+90_{45}$ experiment, the delay δ_1 was set to 2.18 ms, adjusted for a coupling constant $^1J_{CH}$ of 230 Hz, δ_2 was set to 4.00 ms, adjusted for a coupling constant $^1J_{CH}$ of 125 Hz, and δ_3 was set to 3.03 ms, adjusted for a coupling constant $^1J_{CH}$ of 165 Hz.

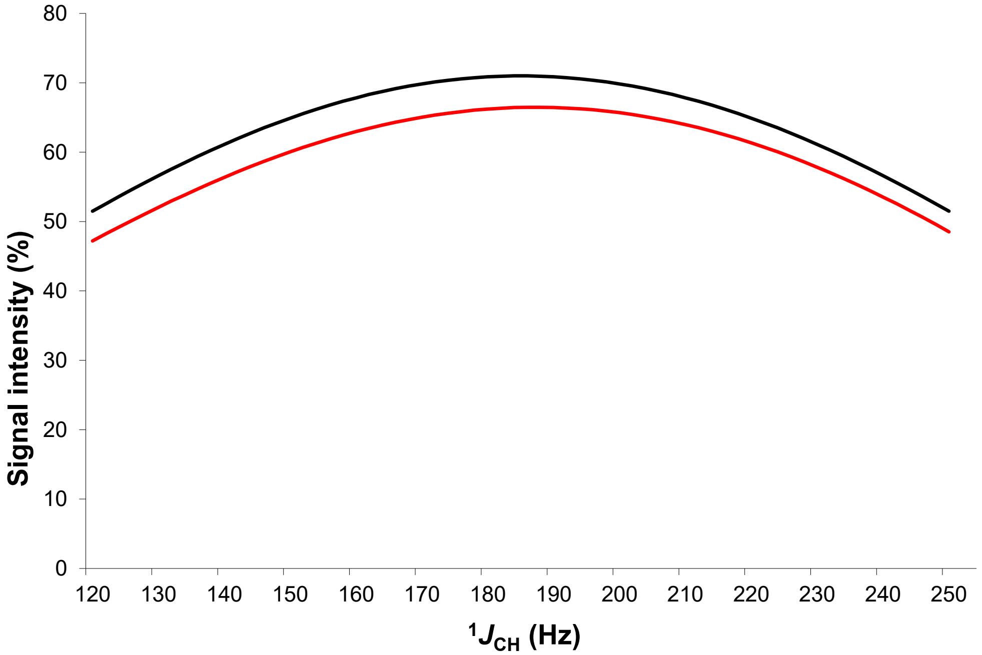

Figure S5. Black line: DEPTQ135$_{90}$, red line: DEPTQ^{+}45$_{90}$. Theoretical amplitude of a CH group as a function of the $^{1}J_{CH}$ coupling constant. The equations used for obtaining the theoretical amplitudes are: 0.71sin^{2} ($\pi J_{CH}\delta$) for DEPTQ and 0.71 sin($\pi J_{CH}\delta_1$)sin($\pi J_{CH}\delta_3$) for DEPTQ^{+}. The $^{1}J_{CH}$ coupling constant range considered for the simulation is [100 – 250 Hz]. For the DEPTQ135$_{90}$ experiment, the delay δ was set to 2.70 ms, adjusted for a coupling constant $^{1}J_{CH}$ of 185 Hz. For the DEPTQ^{+}45$_{90}$ experiment, the delay δ_1 was set to 2.18 ms, adjusted for a coupling constant $^{1}J_{CH}$ of 230 Hz, δ_2 was set to 4.00 ms, adjusted for a coupling constant $^{1}J_{CH}$ of 125 Hz, and δ_3 was set to 2.86 ms, adjusted for a coupling constant $^{1}J_{CH}$ of 175 Hz.

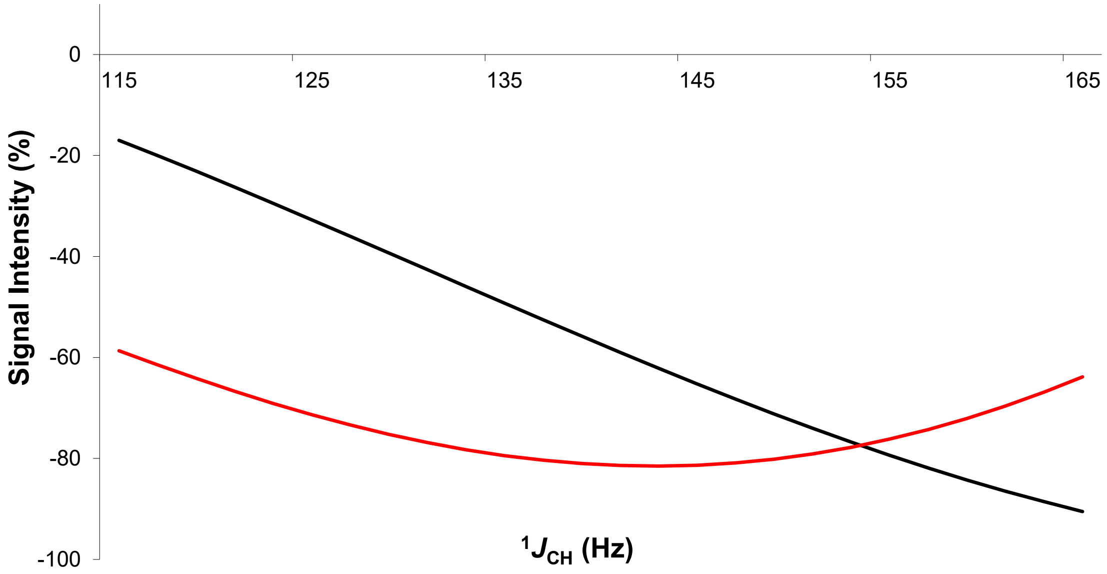

Figure S6. Black line: DEPTQ135$_{90}$, red line: DEPTQ^{+}45$_{90}$. Theoretical amplitude of CH_2 groups as a function of the $^{1}J_{CH}$ coupling constant. The equations used for obtaining the theoretical amplitudes are: 0.35 sin^{2}($2\pi J_{CH}\delta$) -1.00 sin^{4}($\pi J_{CH}\delta$) for DEPTQ and 0.71 sin($\pi J_{CH}\delta_1$)cos($\pi J_{CH}\delta_2$)sin($2\pi J_{CH}\delta_3$) -1.00

sin($\pi J_{CH}\delta_1$)sin($\pi J_{CH}\delta_2$)sin^2($\pi J_{CH}\delta_3$) for DEPTQ$^+$. The $^1J_{CH}$ coupling constant range considered is [110 – 250 Hz], to simulate a molecule possessing all types of $^1J_{CH}$ coupling constants. For the DEPTQ135$_{90}$ experiment, the delay δ was set to 2.70 ms, adjusted for a coupling constant $^1J_{CH}$ of 185 Hz. For the DEPTQ$^+$45$_{90}$ experiment, the delay δ_1 was set to 2.18 ms, adjusted for a coupling constant $^1J_{CH}$ of 230 Hz, δ_2 was set to 4.00 ms, adjusted for a coupling constant $^1J_{CH}$ of 125 Hz, and δ_3 was set to 2.86 ms, adjusted for a coupling constant $^1J_{CH}$ of 175 Hz.

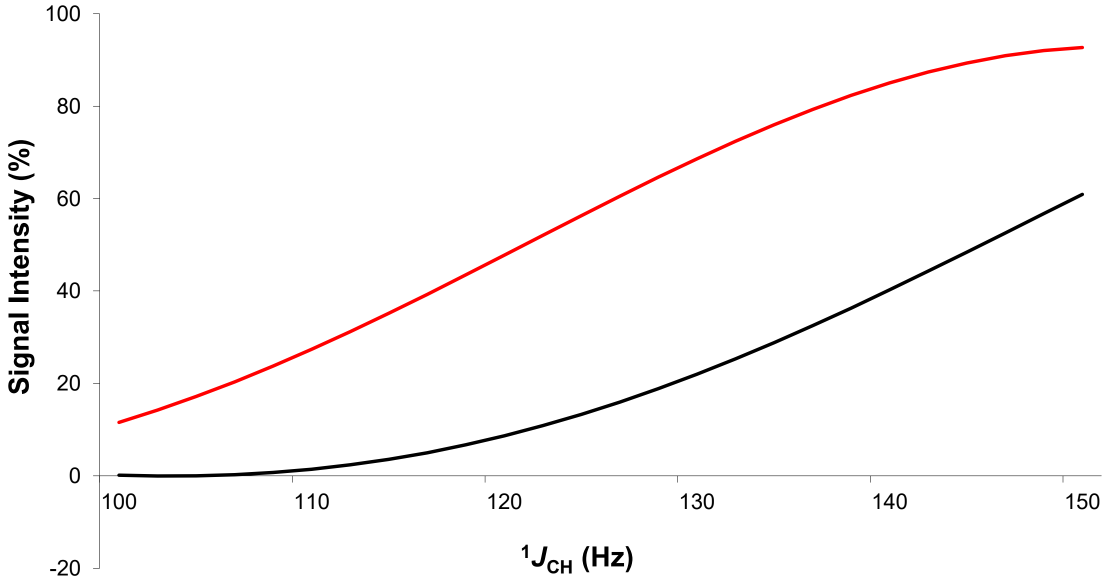

Figure S7. Black line: DEPTQ135$_{90}$, red line: DEPTQ$^+$45$_{90}$.Theoretical amplitude of CH_3 groups as a function of the $^1J_{CH}$ coupling constant. The equations used for obtaining the theoretical amplitudes are: 0.53 sin^2($2\pi J_{CH}\delta$)cos^2($\pi J_{CH}\delta$) – 0.75 sin^2($2\pi J_{CH}\delta$)sin^2($\pi J_{CH}\delta$) + 1.06 sin^6($\pi J_{CH}\delta$) for DEPTQ and 2.11 sin($\pi J_{CH}\delta_1$)cos^2($\pi J_{CH}\delta_2$)sin($\pi J_{CH}\delta_3$)cos^2($\pi J_{CH}\delta_3$) -3 sin($\pi J_{CH}\delta_1$)cos($\pi J_{CH}\delta_2$)sin($\pi J_{CH}\delta_2$)cos($\pi J_{CH}\delta_3$)sin^2($\pi J_{CH}\delta_3$) + 1.06 sin($\pi J_{CH}\delta_1$)sin^2($\pi J_{CH}\delta_2$)sin^3($\pi J_{CH}\delta_3$) for DEPTQ$^+$. The $^1J_{CH}$ coupling constant range considered is [110 – 250 Hz], to simulate a molecule possessing all types of $^1J_{CH}$ coupling constants. For the DEPTQ135$_{90}$ experiment, the delay δ was set to 2.70 ms, adjusted for a coupling constant $^1J_{CH}$ of 185 Hz. For the DEPTQ$^+$45$_{90}$ experiment, the delay δ_1 was set to 2.18 ms, adjusted for a coupling constant $^1J_{CH}$ of 230 Hz, δ_2 was set to 4.00 ms, adjusted for a coupling constant $^1J_{CH}$ of 125 Hz, and δ_3 was set to 2.86 ms, adjusted for a coupling constant $^1J_{CH}$ of 175 Hz.

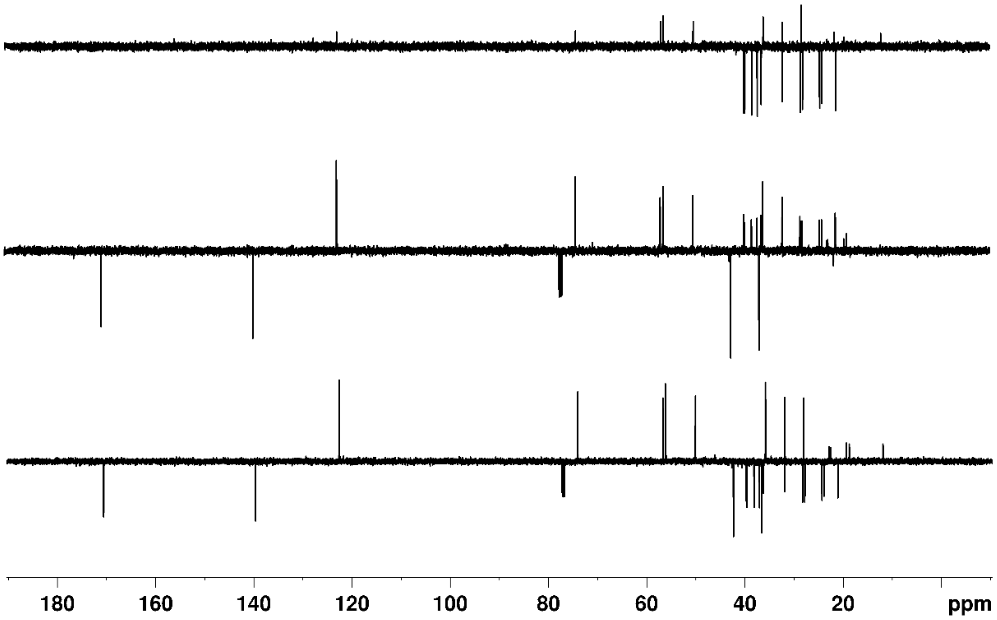

Figure S8. Bottom: DEPTQ135$_{90}$, middle: DEPTQ90$_{45}$ (Cq/CH), top: Difference DEPTQ135$_{90}$ - DEPTQ90$_{45}$ (CH_2/CH_3) spectra of 30 mg of cholesteryl acetate dissolved in 0.7 mL $CDCl_3$. The delay δ was set to 2.70 ms, adjusted for a coupling constant $^1J_{CH}$ of 185 Hz.

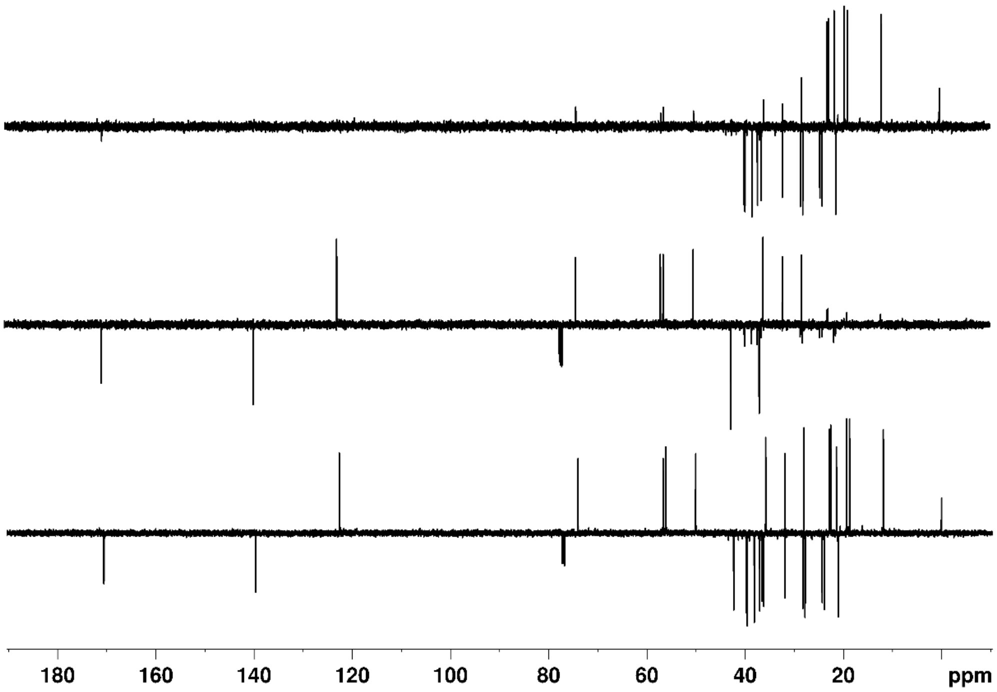

Figure S9. Bottom: DEPTQ⁺45_{90}, middle: DEPTQ⁺90_{45} (Cq/CH), top: Difference DEPTQ⁺45_{90} – DEPTQ⁺90_{45} (CH_2/CH_3) spectra of cholesteryl acetate dissolved in 0.7 mL $CDCl_3$. The delays δ_1, δ_2, and δ_3 were set to, respectively, 2.18 ms, adjusted for a coupling constant $^1J_{CH}$ of 230 Hz, to 4.00 ms, adjusted for a coupling constant $^1J_{CH}$ of 125 Hz, and to 3.03 ms, adjusted for a coupling constant $^1J_{CH}$ of 165 Hz.

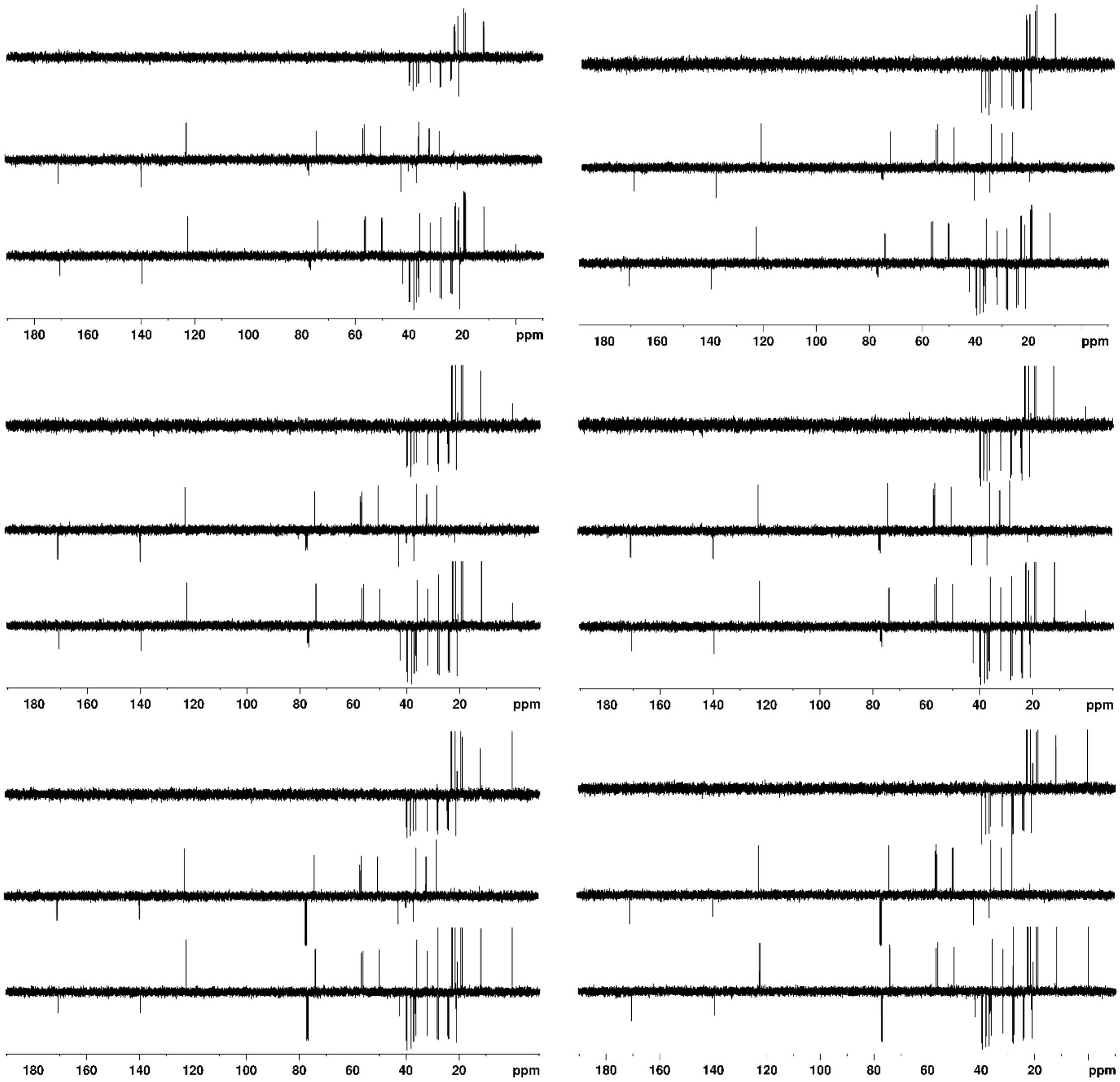

Figure S10. DEPTQ and DEPTQ$^+$ spectra of 30 mg of cholesteryl acetate dissolved in 0.7 mL $CDCl_3$: influence of the relaxation delay. Top: relaxation delay = 2s, middle: relaxation delay = 5s. bottom: relaxation delay = 20s. Left: DEPTQ$^+45_{90}$, DEPTQ$^+90_{45}$, and difference DEPTQ$^+45_{90}$ – DEPTQ$^+90_{45}$ spectra. The delays δ_1, δ_2, and δ_3 were set to, respectively, 3.13 ms, adjusted for a coupling constant $^1J_{CH}$ of 160 Hz, to 4.35 ms, adjusted for a coupling constant $^1J_{CH}$ of 115 Hz, and to 3.45 ms, adjusted for a coupling constant $^1J_{CH}$ of 145 Hz. right: DEPTQ135_{90}, DEPTQ90_{45}, and difference DEPTQ135_{90} – DEPTQ90_{45} spectra. The delay δ was set to 3.45 ms, adjusted for a coupling constant $^1J_{CH}$ of 145 Hz. The spectra were acquired with 64 k data points, leading to an acquisition time of 1.29 s, with a relaxation delay was 2 s, and a NOE building period of 1 s.

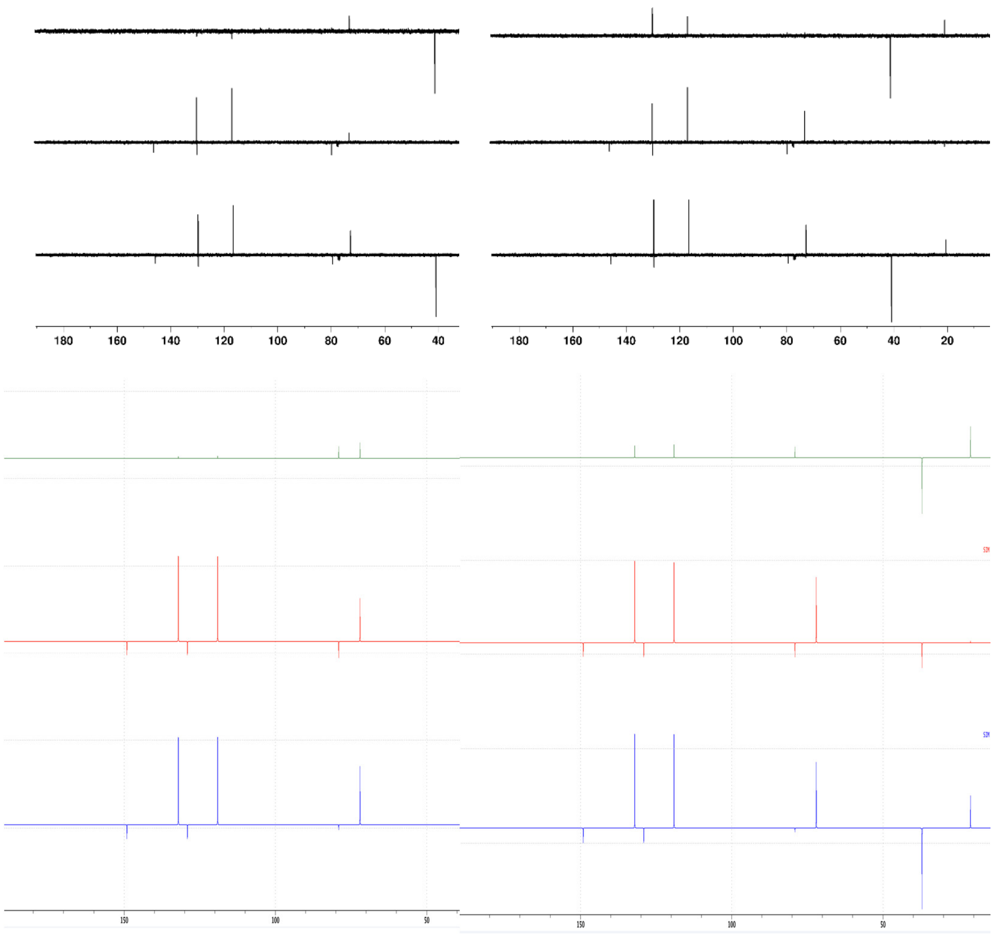

Figure S11. DEPTQ and DEPTQ$^+$ spectra of 30 mg of 4-methyl-*N*,*N*-di(prop-2-yn-1-yl)aniline dissolved in 0.7 mL $CDCl_3$: top: experimental spectra, bottom: simulated spectra using NMRSIM. Right: DEPTQ$^+45_{90}$, DEPTQ$^+90_{45}$, and difference DEPTQ$^+45_{90}$ – DEPTQ$^+90_{45}$ spectra. The delays δ_1, δ_2, and δ_3 were set to 2.18 ms, adjusted for a coupling constant $^1J_{CH}$ of 230 Hz, to 4.00 ms, adjusted for a coupling constant $^1J_{CH}$ of 125 Hz, and to 3.18 ms, adjusted for a coupling constant $^1J_{CH}$ of 165 Hz. respectively. Left: DEPTQ135_{90}, DEPTQ90_{45}, and difference DEPTQ135_{90} – DEPTQ90_{45} spectra. The delay δ was set to 2.70 ms, adjusted for a coupling constant $^1J_{CH}$ of 185 Hz. The spectra were acquired with 64 k data points, leading to an acquisition time of 1.29 s, with a relaxation delay was 2 s, and a NOE building period of 1 s.

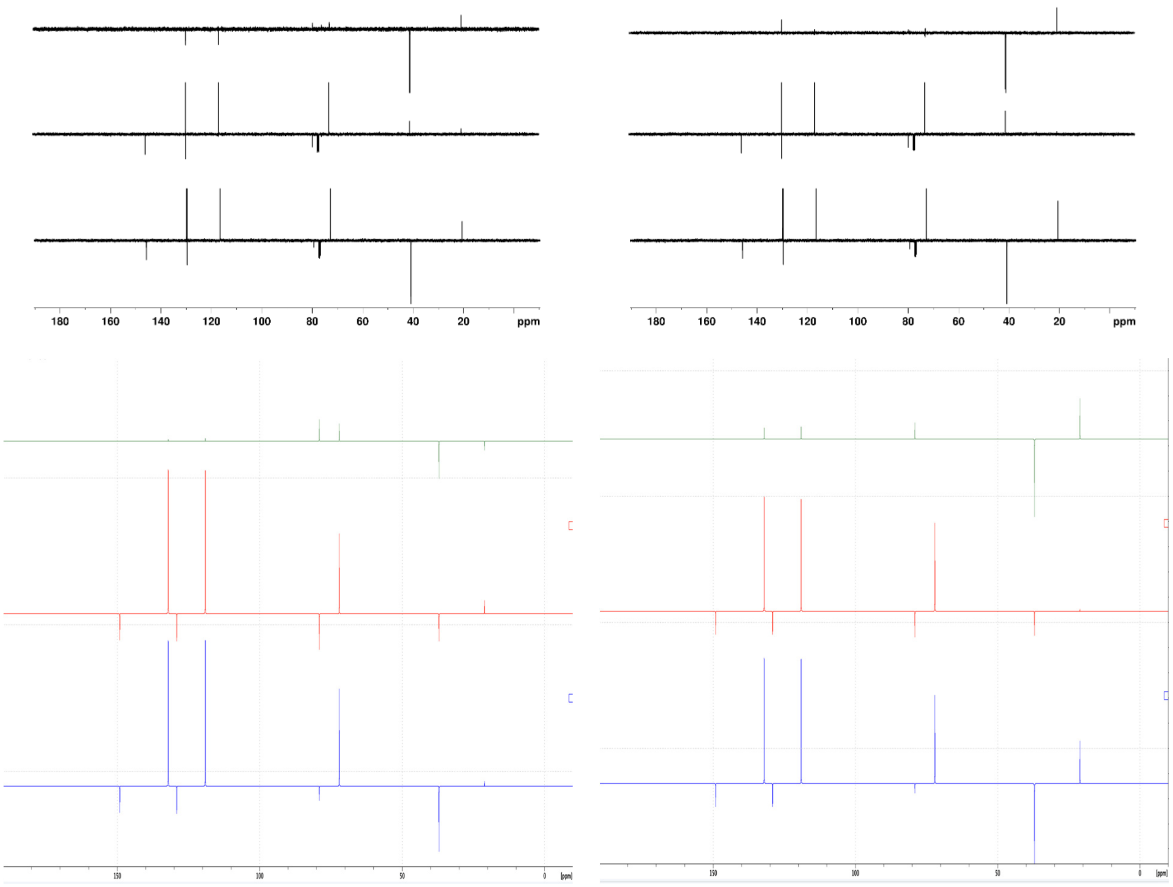

Figure S12. DEPTQ and DEPTQ$^+$ spectra of 30 mg of 4-methyl-*N*,*N*-di(prop-2-yn-1-yl)aniline dissolved in 0.7 mL $CDCl_3$: top: experimental spectra, bottom: simulated spectra using NMRSIM. Right: DEPTQ$^+45_{90}$, DEPTQ$^+90_{45}$, and difference DEPTQ$^+45_{90}$ – DEPTQ$^+90_{45}$ spectra. The delays δ_1, δ_2, and δ_3 were set to 2.18 ms, adjusted for a coupling constant $^1J_{CH}$ of 230 Hz, to 4.00 ms, adjusted for a coupling constant $^1J_{CH}$ of 125 Hz, and to 3.18 ms, adjusted for a coupling constant $^1J_{CH}$ of 165 Hz. respectively. Left: DEPTQ135_{90}, DEPTQ90_{45}, and difference DEPTQ135_{90} – DEPTQ90_{45} spectra. The delay δ was set to 2.70 ms, adjusted for a coupling constant $^1J_{CH}$ of 185 Hz. The spectra were acquired with 64 k data points, leading to an acquisition time of 1.29 s, with a relaxation delay was 20 s, and a NOE building period of 1 s.
